# Identification and validation of differentially expressed disulfidptosis-related genes in hypertrophic cardiomyopathy

**DOI:** 10.1186/s10020-024-01024-1

**Published:** 2024-12-19

**Authors:** Huimin Fan, Xin Tan, Shuai Xu, Yiyao Zeng, Hailong Zhang, Tong Shao, Runze Zhao, Peng Zhou, Xiaohong Bo, Jili Fan, Yangjun Fu, Xulong Ding, Yafeng Zhou

**Affiliations:** 1 Center of Translational Medicine and Clinical Laboratory, Suzhou Dushu Lake Hospital, The Fourth Affiliated Hospital to Soochow University, Suzhou, 215000 China; 2 Department of Cardiology, Suzhou Dushu Lake Hospital, The Fourth Affiliated Hospital of Soochow University, Medical Center of Soochow University, Suzhou, 215000 China; 3 Institute for Hypertension, Soochow University, Suzhou, 215000 China; 4 Department of Cardiovascular Disease, Taihe County People’s Hospital, Fuyang, 236600 China; 5 Department of Neurology, The Third People’s Hospital of Hefei, Hefei City, Anhui Province 230041 China

## Abstract

Hypertrophic cardiomyopathy (HCM) is one of the most common cardiovascular diseases with no effective treatment due to its complex pathogenesis. A novel cell death, disulfidptosis, has been extensively studied in the cancer field but rarely in cardiovascular diseases. This study revealed the potential relationship between disulfidptosis and hypertrophic cardiomyopathy and put forward a predictive model containing disulfidptosis-associated genes (DRGs) of GYS1, MYH10, PDMIL1, SLC3A2, CAPZB, showing excellent performance by SVM machine learning model. The results were further validated by western blot, RNA sequencing and immunohistochemistry in a TAC mice model. In addition, resveratrol was selected as a therapeutic drug targeting core genes using the CTD database. In summary, this study provides new perspectives for exploring disulfidptosis-related biomarkers and potential therapeutic targets for hypertrophic cardiomyopathy.

**Supplementary Information:**

The online version contains supplementary material available at 10.1186/s10020-024-01024-1.

## Introduction

Hypertrophic cardiomyopathy (HCM) is the most common heritable cardiovascular disorder characterized by left ventricular hypertrophy (LVH), usually without the left ventricular (LV) cavity enlargement (Marian and Braunwald 2017). The clinical manifestations of HCM are highly heterogeneous, with primary symptoms including chest pain, dyspnea, palpitation, and syncope (Tuohy et al. 2020), and it is an important cause of arrhythmical sudden cardiac death, heart failure and atrial fibrillation in young people. Globally, the incidence rate of HCM is over 1/500 (Jia et al. 2019), affecting both males and females(Veselka et al. 2021). At present, the treatment of hypertrophic cardiomyopathy mainly aims at alleviating symptoms, reducing complications, and preventing sudden death. The therapeutic drugs for HCM are limited, and these drugs neither can prevent the disease from progressing, nor can they effectively reduce the incidence of complications. One particular roadblock is the limited understanding of particular mechanisms in HCM.

Regulated cell death programs, including apoptosis, necroptosis, cuproptosis, ferroptosis, and others, play an essential role in the occurrence and development of cardiovascular diseases (Chen et al. 2023a, b; Amgalan et al. 2017; Del Re et al. 2019; Fang et al. 2020, 2023; Weng et al. 2023). In 2023, Liu et al. (Liu et al. 2023) proposed a new form of cell death called disulfidptosis. Under the condition of glucose starvation, disulfide accumulates continuously in cells with high expression of solute carrier family 7 member A11 (SLC7A11), inducing disulfide stress and actin collapse, ultimately destroying cytoskeleton structure and leading to cell death. Moreover, inhibitors of ferroptosis, apoptosis, necrosis and autophagy could not protect cells from glucose starvation-induced death, highlighting the uniqueness of disulfidptosis(Liu et al. 2023).

Disulfidptosis has been mainly studied in cancer. Recently it was reported that loss of cardiac Ferritin H could facilitate cardiomyopathy via SLC7A11, a crucial protein in disulfidptosis, mediated ferroptosis, indicating the potential role of disulfidptosis in the development of cardiomyopathy (Fang et al. 2020). Another study revealed that in lysosomal storage diseases, autophagic cell death could promote hypertrophic cardiomyopathy, demonstrating the relationship between cell death and HCM (Rabinovich-Nikitin and Kirshenbaum 2021). However, the pathogenic genes of HCM are not fully clarified and the role of disulfidptosis-related genes (DRGs) in HCM has not been explored yet.

In this study, we used GSE36961 as the training dataset, GSE141910 and GSE160997 as validation datasets to develop and validate an HCM prediction model based on DRGs. Furthermore, immune infiltration analysis revealed the relationship between DRGs-related immune processes and HCM. A completing endogenous RNA (ceRNA) network and drug prediction model targeting the identified DRGs were also established. In addition, in vivo HCM model further confirmed our findings. These results identified a predictive model and therapeutic targets for HCM.

## Materials and methods

### Data collection and preparation

GSE36961 (a dataset of gene expressions and clinical data of 37 controls and 110 HCM patients), GSE141910 (a dataset of gene expressions and clinical data of 162 controls and 29 HCM patients) (Tan et al. 2020) and GSE160997 (a dataset of gene expressions of 5 controls and 18 HCM patients) (Maron et al. 2021) were from the Gene Expression Omnibus (GEO) database. Among these, GSE36961 was used as a training dataset, GSE141910 and GSE160997 were merged as a validating dataset. All the datasets analysis were conducted using R software (version 4.4.0) unless otherwise specified, with differentially expressed genes (DEGs) were determined using the limma package (Ritchie et al. 2015) and Heatmaps were generated using the heatmap package. The correlation analysis of differentially expressed DRGs were performed using the cor function in R 4.3.2.

### Cluster analysis based on DRG expressions

Consistent cluster analysis was performed using the Consensus Cluster Plus package (Wilkerson and Hayes 2010) to identify different DRG-related clusters. The optimal k value was determined according to the proportion of ambiguous clustering (PAC). |log2 FC| > 1 and FDR < 0.05 as cut-off values between two DRG clusters.

### Establishment of predictive model of HCM based on DRGs

A computational framework that combined four machine learning algorithms was adopted to construct a predictive model of HCM. The machine learning algorithms included random forest (RF) (Asadi et al. 2021), extreme gradient boosting (XGB) (Kanda et al. 2022), generalized linear model (GLM) (Mahmoudi et al. 2023) and support vector machine (SVM) (Gold et al. 2005). The distribution of model residuals among four machine learning methods was visualized with the DALEX package. The Area Under the Curve (AUC) of the Receiver Operating Characteristic (ROC) curves was visualized using the pROC package (Wilkerson and Hayes 2010). The optimal machine learning method was selected according to the AUC of ROC and the five top genes were identified as core diagnostic genes.

### Construction and validation of nomogram

A nomogram was constructed using the RMS R package (Harrell 2023) to validate and operationalize the predictive capability of the models. Decision Curve Analysis (DCA) and calibration curves were employed to evaluate the predictive performance of the nomogram.

### Analysis of immune cell infiltration in HCM patients

The infiltration of immune cells was evaluated with the CIBERSORT website (https://cibersort.stanford.edu/), which is an analytical tool from the Alizadeh Lab and Newman Lab to impute gene expression profiles and provide an estimation of the abundances of member cell types in a mixed cell population, using gene expression data. Significance was established when p-values were less than 0.05, and the sum of.

the proportions of all 22 immune cell types in each sample equaled one.

### Establishment of ceRNA network

The lncRNA-miRNA-mRNA ceRNA network was established using MiRDB (https://www.mirdb.org/) (Chen and Wang 2020) to reverse the prediction of miRNAs corresponding to the validated hub genes. The binding site and target genes of miRNAs were predicted by TargetScan database (Agarwal et al. 2015) (https://www.targetscan.org/) and miRanda database. Prediction of miRNA-lncRNA interactions was achieved from the SpongeScan database (Furió-Tarí et al. 2016) (http://spongescan.rc.ufl.edu). The ceRNA network that integrated mRNAs, miRNAs, and lncRNAs was graphically represented using Cytoscape software (version 3.7.2).

### Evaluation of candidate small-molecule drugs

Potential therapeutic drugs targeting the core DRGs were acquired from the CTD database (http://ctdbase.org/) (Davis et al. 2021). A drug-gene network was established by Cytoscape software (3.7.2). Molecular docking of candidate drugs and core genes was performed using Autodock Vina software V1.1.2 and visualized in Pymol V2.0.0.

### HCM mouse model established by transverse aortic constriction (TAC)

6–8 weeks male C57 mice were purchased from Beijing Vital River Company. Mice were housed at a moderate temperature (22 ± 2 °C), appropriate humidity (55 ± 5%), a 12-hour light/dark cycle, and free access to food and water. Mice were acclimated to the circumstance for one week prior to all experiments and randomly divided into different groups (*n* = 3–5). All animal experiments were performed with the approval of the Institutional Animal Care and Use Committee of the Fourth Affiliated Hospital of Soochow University (2400321).

A HCM mouse model was used, as described previously (Guo et al. 2015). Briefly, male C57BL/6 mice were anesthetized with pentobarbital sodium with a dose of 50 mg/kg. They were fixed on a heating pad in a supine position to maintain body temperature. The neck and chest were depilated with an animal shaving machine and disinfected with 75% ethanol. After tracheal intubation, the breathing frequency of the mice was approximately 120 times per minute. The skin, pectoral muscle and intercostal muscle were cut from the second intercostal space in the heart and then the chest was enlarged. Microscopic tweezers were used to separate thymus and expose aortic arch. A short section of 6.0 suture silk thread was placed under the aortic arch between the innominate artery and the left common carotid artery, and a slipknot was tied around the aortic arch. A flat-headed needle was inserted into the slipknot and placed parallel to the artery, and the slipknot was used to fasten the needle and the artery. Then the needle was quickly taken out to obtain the surgical stenosis of the aortic arch at the distal end of the innominate artery with a theoretical diameter of 0.4 mm. The thymus was reset, and the intercostal muscle and skin were sutured with 6.0 polypropylene suture. Then 0.2 ml penicillin was injected intraperitoneally. The operation for sham group is the same as the TAC group except that the aorta is not ligated. TAC causes moderate or severe mechanical obstruction of the left ventricular outflow, and evident left ventricular cardiac hypertrophy can be formed after 4 weeks.

All animal experiments were approved by the Animal Care and Use Committee of The Fourth Affiliated Hospital of Soochow University, Suzhou Dushu Lake Hospital.

### Cardiac echocardiography

The mice were anesthetized with 3% isoflurane (Baxter International, USA) and fixed on the operating table. Ultrasound scanning (FUJIFILM VisualSonics, Canada) was performed to obtain cardiac parameters. Specific parameters for HCM include interventricular ventricular septum (diastole) (IVSD), interventricular ventricular septum (systole) (IVSS), left ventricular mass (LV mass), ejection fraction (EF) and fractional shortening (FS). Statistical analysis was conducted using the average values of three cardiac cycles.

### RNA extraction and RNA-sequencing of mice heart tissue

Grinded mice heart tissue total RNA was extracted using RNA-Quick Purification Kit (ES Science) according to the manufacture’s instruction. Then 500 ng RNA was used to prepare RNA-seq libraries with NEB Next Ultra II Direcrional RNA library Prep Kit (NEB, cat# E7760L). Equal quantities of cDNA were mixed for next sequencing (GENEWIZ, Suzhou, China).

### Protein extraction from mouse heart tissue and western blot

After the mice’s hearts were taken out, appropriate volume of RIPA lysate (Beyotime) was added and ground into tissue suspension in a grinder. The tissue suspension was lysed on ice for 40 min, followed by centrifugation at 13,000 rpm for 15 min. The supernatant was added with loading buffer (TAKARA) and boiled in a 100 ℃ metal bath for 10 min.

An equal amount of protein samples was electrophoresed with SDS-PAGE gels (Epizyme Biotechnology) and then transferred onto a 0.45 μm PVDF membrane (Millipore). Blots were then blocked in 5% non-fat milk (Solarbio) at room temperature for 1 h, then incubated with specific primary antibodies at 4℃ overnight: Anti-GYS1 (ProteinTech, 1:1000), Anti-MYH10 (ProteinTech, 1:1000). Anti-SLC3A2 (ProteinTech, 1:1000), anti-CAPZB (ProteinTech, 1:1000). Anti-PDLIM1 (ProteinTech, 1:1000). The next day, Blots were washed with 1×TBST for 15 min, 3 times, then incubated with HRP-linked secondary antibody (1:10000, CST) at room temperature for 1 h. The membranes were scanned with the ChemiDoc XRS+ (Bio-Rad, USA) and analyzed with Image Lab software.

### Immunohistochemistry (IHC) for mouse heart tissue

Heart tissues collected from control and HCM model mice were fixed with formalin, embedded with paraffin and cut into 4 μm pieces. The slices were fixed with 10% neutral buffered formalin at room temperature for 10 min, rinsed in frozen acetone at -20 ℃ for 10 min and dry. Then rinsed in methanol at -20℃ for 10 min, fixed with 3% formaldehyde at room temperature for 15 min and then fixed in methanol solution at -20 ℃ for 5 min. The slices were washed with 1xPBS twice, 5 min each time. Dewaxing: quickly put the slices into xylene for 2 times, 5 min each. Gradient hydration: put slices in 100%, 90%, 80% and 70% ethanol for 5 min, and put them in distilled water for 5 min. Endogenous peroxidase blocking: put slices in a wet box with 3% hydrogen peroxide at room temperature for 10 min, then put them in distilled water for 1 min. Antigen repair: put in 65 ℃ water bath for 10 min, and cool it to room temperature. Permeate with 0.3% Triton at room temperature for 15 min. Primary antibody: Add 100 µL Anti-GYS1 (ProteinTech, 1:100), Anti-MYH10 (ProteinTech, 1:100). Anti-SLC3A2 (ProteinTech, 1:100), anti-CAPZB (ProteinTech, 1:100). Anti-PDLIM1 (ProteinTech, 1:100), incubate at 4 ℃ overnight. The next day, the slices were washed with PBS buffer for 3 min×3 times. The secondary antibody was incubated at room temperature for 30 min. Add freshly prepared DAB chromogenic solution and incubate at room temperature for 5 ~ 8 min. Rinse with tap water and recolor with hematoxylin dye solution. Then, dehydrate with ethanol, transfer to xylene for 15 min. Neutral gum was dropped on the tissue and cover with a glass cover. After 24 h, they were observed and photographed under a microscope.

### Hematoxylin Eosin (HE) staining

Heart tissues collected from control and HCM model mice were fixed with formalin, embedded with paraffin and cut into 4 μm pieces. Put paraffin slices into xylene I 10 min, xylene II 10 min, xylene III 10 min, anhydrous ethanol I 5 min, anhydrous ethanol II 5 min, 90% alcohol 5 min, 80% alcohol 5 min, 70% alcohol 5 min, 50% alcohol 5 min for gradient dewaxing. Slices were stained with hematoxylin for 0.5–1 min, rinsed with tap water, differentiated with 1% hydrochloric acid alcohol for several seconds, rinsed with tap water, then 1% ammonia water solution turned blue for 1 min, rinsed with running water for several seconds, dyed with eosin dye solution for several seconds, and rinsed with running water.

### MASSON staining

Mice heart tissue paraffin slices were put in xylene I 5 min, xylene II 5 min, xylene III 5 min, anhydrous ethanol 1 min, 95% ethanol 1 min, 75% ethanol 1 min, and washed with tap water for several seconds. Then they were stained with the prepared Weigert iron hematoxylin staining solution for 8 min. Slices were differentiated for 15 s with acidic ethanol differentiation solution and washed with water. Slices stained with masson bluing solution returned to blue for 5 min and were washed with distilled water for 1 min. Then, they were stained with Fuchsin Staining Solution for 5 min and washed with weak acid working solution for 1 min. Slices were washed with phosphomolybdic acid solution for 1 min and weak acid working solution for 1 min. Dyeing with aniline blue dye solution for 2 min and weakly pickling for 1 min. Dewatering transparency: slices were dehydrated quickly in 95% ethanol for 2–3 s, anhydrous ethanol for 5–10 s, 3 times, xylene for 1–2 min, 3 times, and sealed with neutral gum.

### Statistics

Statistical analysis was performed using GraphPad Prism 7 software. Results were presented as mean ± SD of 3 to 6 parallel experiments. Unpaired Student’s t-test was used for comparisons between two groups after normal distribution. *p < 0.05, **p < 0.01, *ns* (non-significant).

## Results

### DRGs expression and immune infiltration analysis of HCM patients

**Fig. 1:**
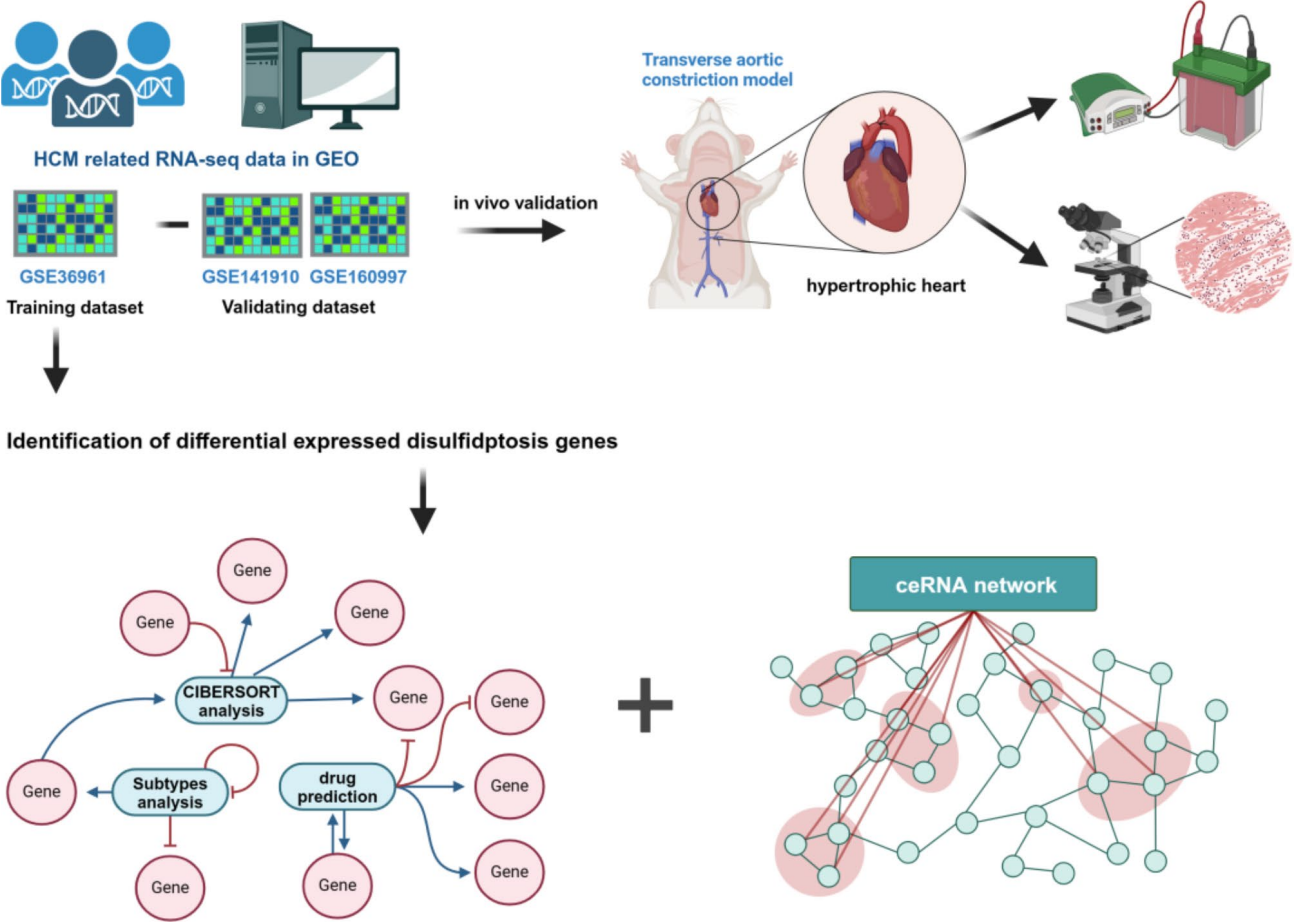
Schematic diagram of this study. The figure is created with BioRender.com

**Fig. 2:**
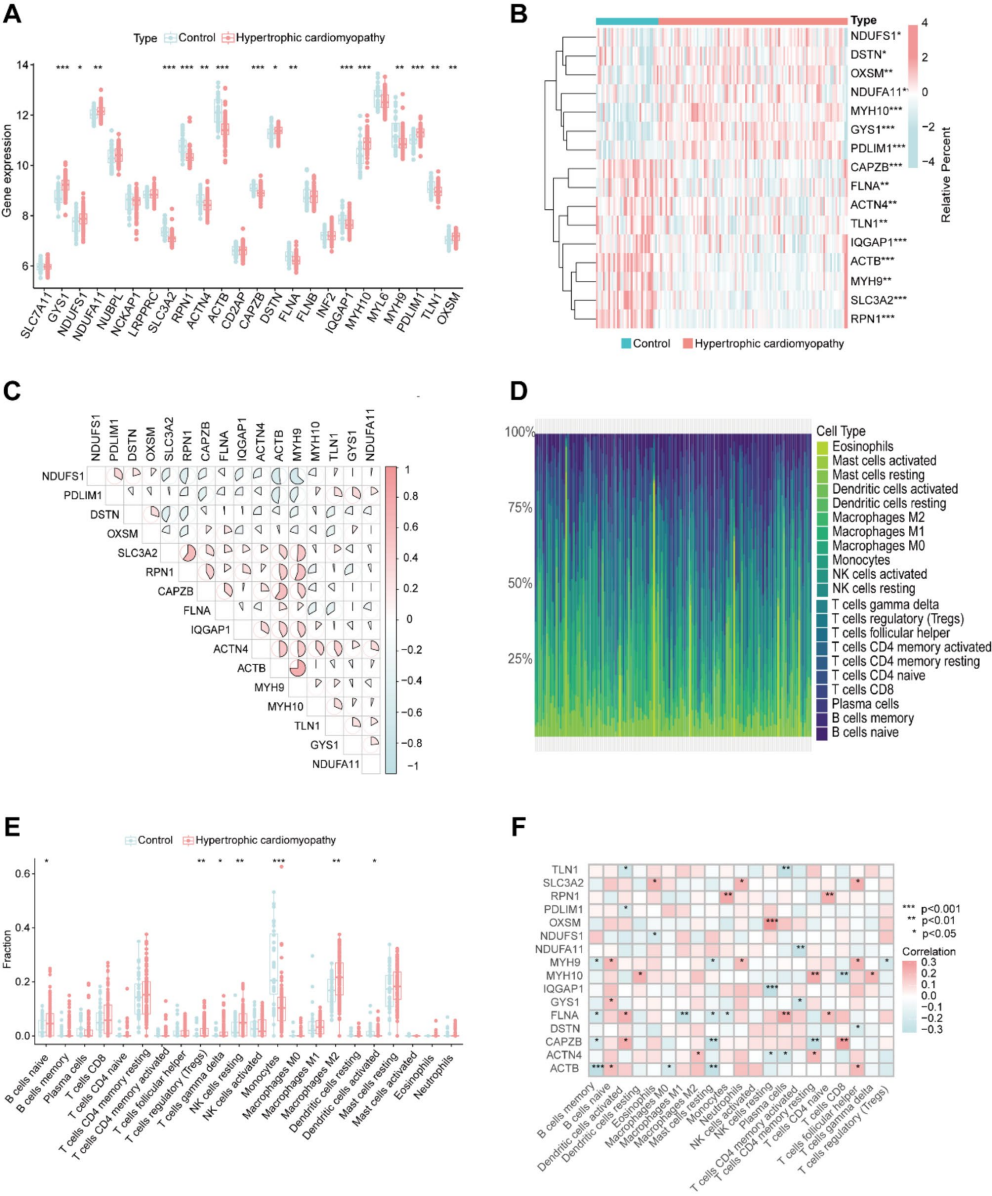
Identification of DRG expressions and immune analysis in HCM patients (**A**) The expression pattern of DRGs in control people and HCM patients. (**B**) Heatmap of 16 differentially expressed DRGs between control and HCM patients. (**C**) The correlation pattern of 16 differentially expressed DRGs. (**D**) Heatmap of immune cell infiltration of control people and HCM patients. (**E**) The immune cell infiltration pattern of control people and HCM patients. (**F**) The correlation analysis between 16 DRGs and immune cells. * p< *0.05*, ** p< *0.01*

To investigate the relationship between HCM and disulfidptosis, we designed the corresponding experimental workflow, as illustrated in Fig. 1. First, we selected 24 DRGs based on previous literatures (Liu et al. 2023; Machesky 2023) and analyzed the difference in the expression of DRGs between healthy people and HCM patients in GSE36961 dataset. Comparative analysis revealed increased expression levels on GYS1, NDUFS1, NDUFA11, DSTN, MYH10, PDLIM1 and OXSM, along with decreased expression levels on SLC3A2, RNP1, ACTIN4, ACTB, CAPZB, FLNA, IQGAP1, MYH9 and TLN1 (Fig. 2A and B). In addition, we conducted a thorough correlation analysis of differentially expressed DRGs to explore their potential roles in HCM. Notably, some DRGs showed significant positive correlations, such as ACTB and MYH9, SLC3A2 and RPN1, CAPZB and ACTB. At the same time, negative correlations also existed between these DRGs such as NDUFS1 and MYH9, NDUFS1 and ACTB (Fig. 2C).

Additionally, an immune infiltration analysis was performed to explore the difference of immune infiltration between HCM patients and healthy people (Fig. 2D). Among the total 22 immune cell types, 7 immune cells showed altered infiltration patterns in HCM patients, including increased infiltration of naïve B cells, regulatory T cells (Tregs), gama delta T cells, resting NK cells and M2 macrophages, and decreased infiltration of monocytes and activated dendritic cells (Fig. 2E). Furthermore, correlation analysis revealed disulfidptosis in naïve B cells, activated dendritic cells, neutrophils, activated NK cells, naïve CD4+ T cells and CD8+ T cells (Fig. 2F). These findings suggested that the potential role of disulfidptosis and immune cell infiltration as important etiological factors in HCM patients

### Identification of HCM disulfidptosis cluster

**Fig. 3:**
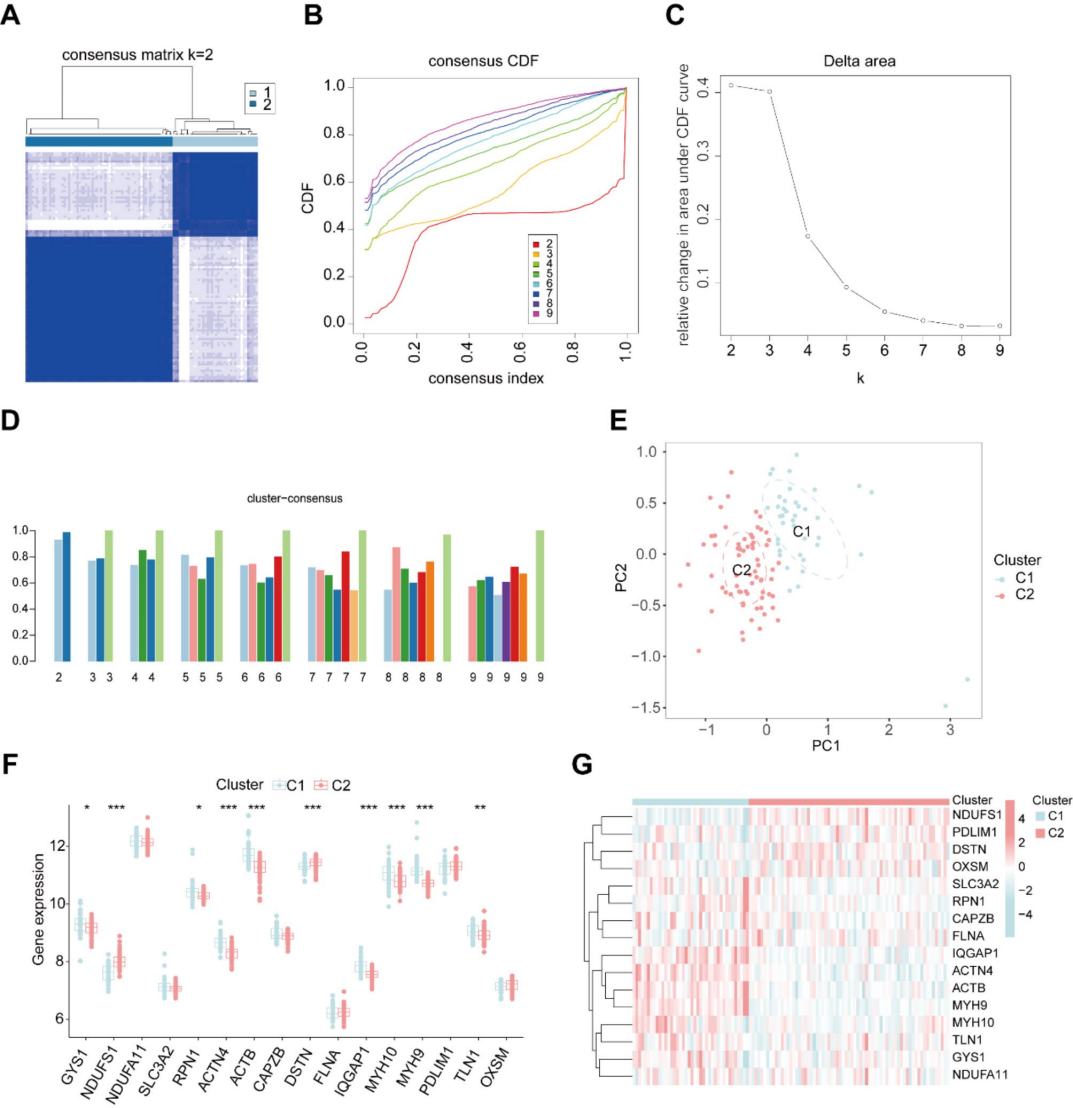
Identification of disulfidptosis-related clusters in HCM. (**A**) Consensus clustering matrix when k = 2. (**B**, **C**) Representative cumulative distribution function (CDF) curves (**B**), CDF delta area curves (**C**). (**D**) The score of consensus clustering. (**E**) The distribution of two clusters. (**F**) The different expressions of 16 DRGs between cluster 1 and cluster 2. (**G**) The expression pattern of 16 DRGs is presented in the heatmap

To better understand the expression pattern of DRGs in HCM patients, consensus cluster analysis was conducted for 16 differentially expressed DRGs. The consensus index exhibited a stable fluctuation within the range of 0.2–0.6 (Fig. 3A and B). By varying the cluster number (k) from 2 to 9, the area under the CDF curve depicted significant differences between clusters (k and k-1) (Fig. 3C). As illustrated in Fig. 3D, a high consistency score (> 0.9) was observed only when k = 2. Based on the consensus matrix heatmap, we divided the 109 patients into two distinct clusters, denoted as cluster 1 (*n* = 40) and cluster 2 (*n* = 69) (Fig. 3E). DRGs expression demonstrated pronounced differences between the two clusters. Cluster 1 exhibited increased expressions of GYS1, RPN1, ACTN4, ACTB, IQGAP1, MYH10, MYH9 and TLN1, while cluster 2 had elevated expression of NDUFS1 and DSTN (Fig. 3F and G).

Furthermore, immune cell infiltration analysis was conducted between the two clusters, which showed elevated infiltration of follicular helper T cells, M2 macrophages, neutrophils and reduced infiltration of resting mast cells in cluster 1 compared with cluster 2 (Figures S1 A and B). Gene Ontology (GO) analysis of differentially expressed genes indicated that several cellular processes up- or down-regulated in cluster 2 compared to cluster 1. Up-regulated processes included pathways related to the cytoskeleton, such as postsynaptic cytoskeleton organization, myosin complex, actin cytoskeleton, and collagen fibril organization. While down-regulated processes were correlated with cell metabolism, such as carboxylic ester hydrolase activity, long chain fatty acid metabolic process, fatty acid deravitive binding. (Figure S2 A). In addition, KEGG pathway enrichment showed distinct biological processes between the two clusters. Cluster 1 was enriched in cardiac muscle contraction, one carbon pool by folate, biosynthesis of unsaturated fatty acids, folate biosynthesis and so on. While cluster 2 was enriched in tight junction, focal adhesion, regulation of actin cytoskeleton, and ECM receptor interaction (Figure S2 B). These differences in gene expression and signaling pathways revealed distinct biological features between the two groups of HCM patients.

### Construction and evaluation of machine learning models

**Fig. 4:**
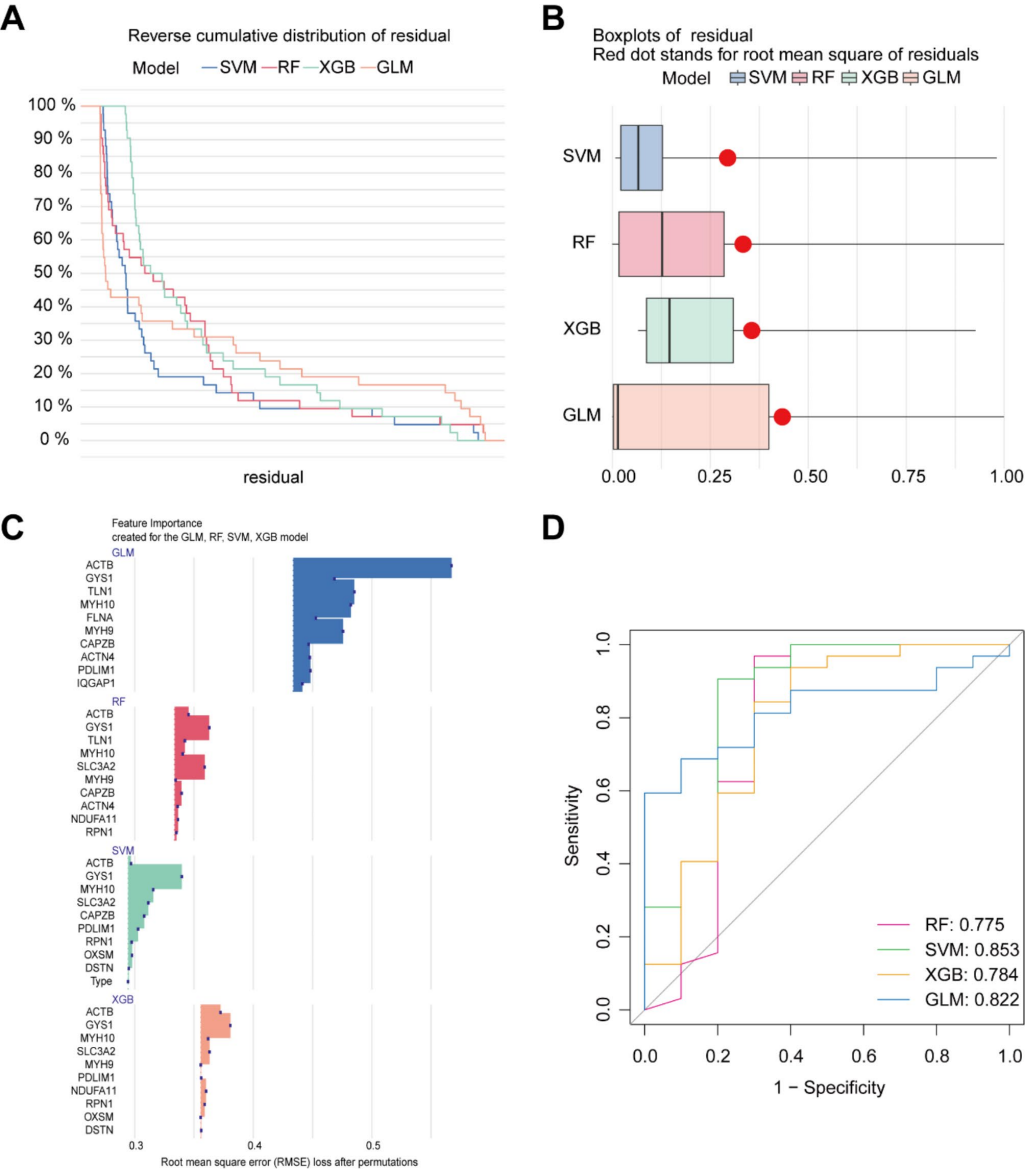
Construction and evaluation of four machine learning models. (**A**) Cumulative residual distribution of SVM, RF, XGB, GLM models. (**B**) Boxplots representing the residuals of each machine learning model. (**C**) Feature importance of top 10 DRGs in each machine learning model. (**D**) Receiver Operating Characteristic (ROC) analysis of four machine learning models

To identify specific diagnostic markers for HCM, we employed four machine-learning models: SVM, RF, GLM and XGB. Residual visualization for each model in the training dataset was accomplished by model interpretation using the ‘DALEX’ software package. By comparing residual, root mean squared error (RMSE) and diagnostic performance of four models, we found the SVM model exhibited relatively minor residual differences (Fig. 4A and B) and RMSE (Fig. 4C), as well as the optimal ROC value (SVM:0.853, GLM:0.822, XGB:0.784, RF:0.775) (Fig. 4D). So we selected the top five genes (GYS1, MYH10, SLC3A2, CAPZB and PDLIM1) from the SVM model as the key predictors for subsequent analysis.

**Fig. 5:**
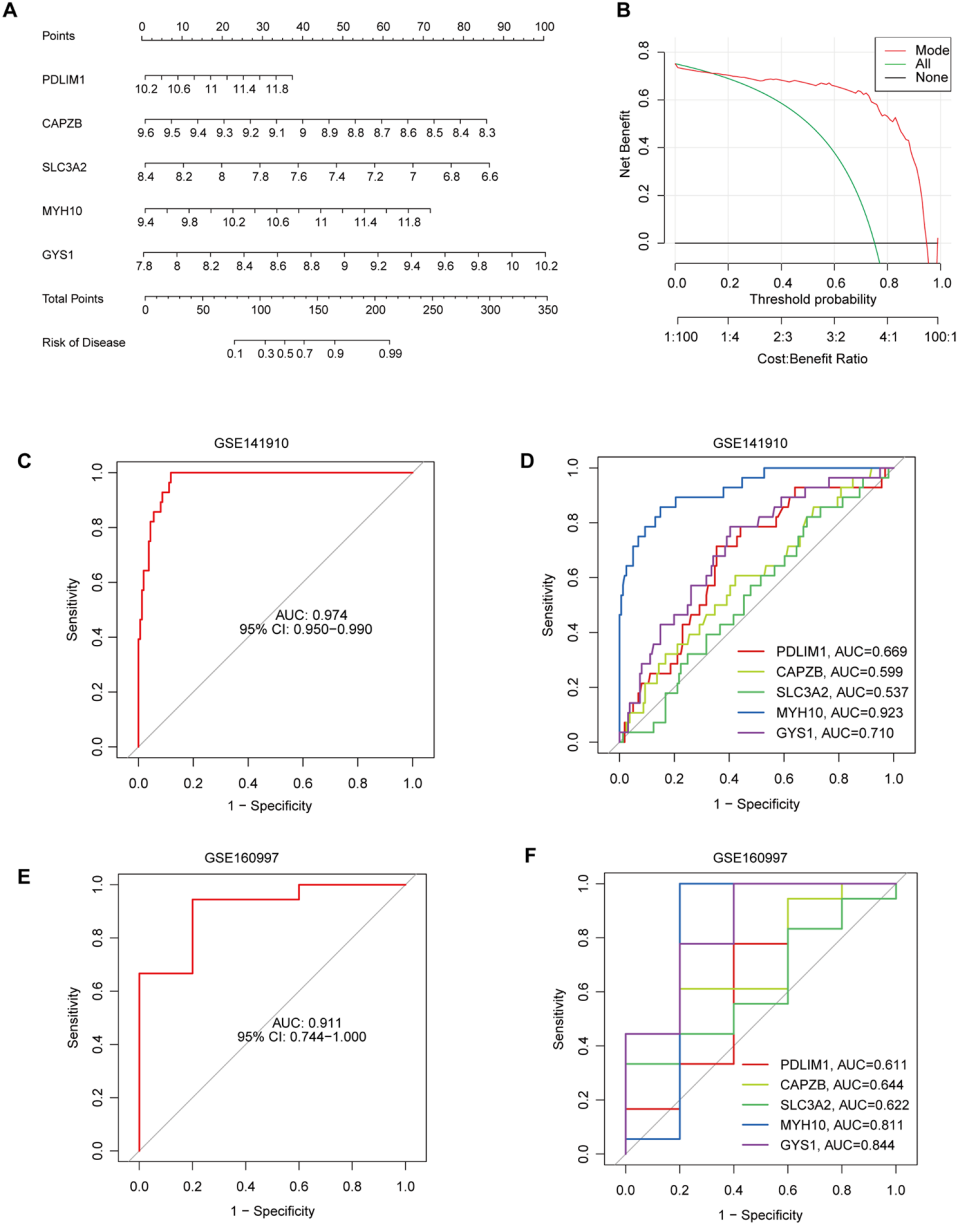
Validation of the 5-gene-based SVM model. (**A**) Construction of a nomogram for predicting the risk of HCM based on the 5-gene-based SVM model. (**B**) Calibration curve for assessing the predictive efficiency of the nomogram model. (**C**, **D**) ROC analysis of the prediction nomogram based on 5-fold cross-validation in validation dataset GSE141910. (**E**, **F**) ROC analysis of the prediction nomogram based on 5-fold cross-validation in validation dataset GSE160997

Next, a nomogram was constructed to evaluate the predictive efficiency of the SVM model using 110 HCM cases in the training dataset (Fig. 5A). Calibration curves and DCA were employed to assess the prediction accuracy of the nomograms. The calibration curve demonstrated minimal error between the actual and predicted risks in the HCM cluster (Fig. 5B). The predictive capability of core biomarkers was verified using the validation datasets GSE141910 and GSE160997. The ROC curves showed that the predictive model containing the five core markers performed well with AUCs of 0.974 (GSE141910) and 0.911 (GSE160997) (Fig. 5C-F). Notably, MYH10 stood out for its best performance with AUCs among all five genes in GES141910 dataset (Fig. 5D), and ranked second in GSE160997 dataset (Fig. 5F). These results indicated the effectiveness of the diagnostic model in distinguishing HCM patients from normal people.

### CeRNA network establishment and drug prediction

**Fig. 6:**
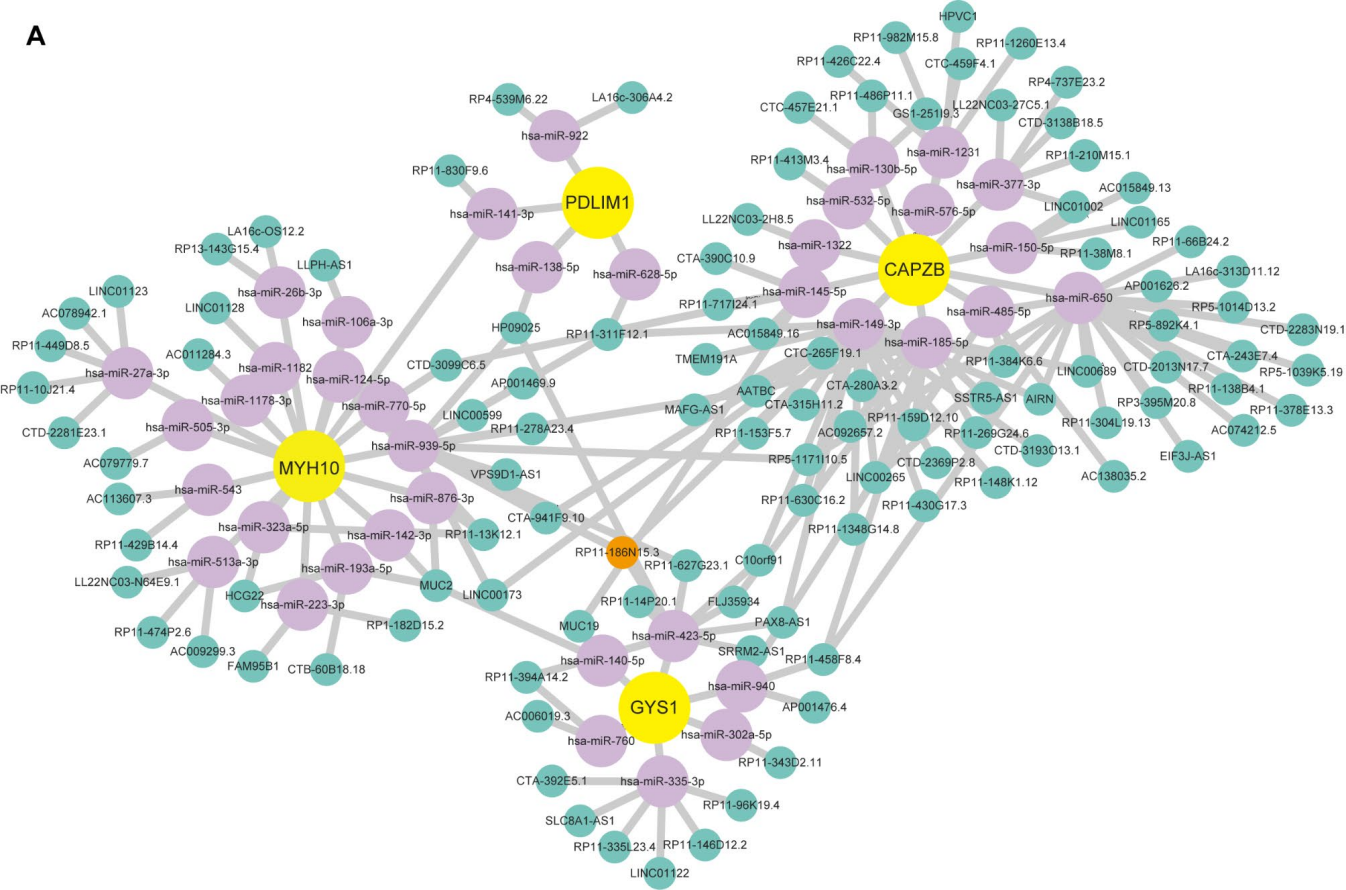
CeRNA network based on five-core DRGs. The yellow nodes represent mRNAs, purple nodes represent miRNAs and green nodes represent lncRNAs

To better understand the regulation mechanisms of these key genes, we constructed a ceRNA network using databases including miRanda, targetScan, miRDB and SpongeScan. This network comprises 153 nodes, including 4 core diagnostic markers (SLC3A2 could not be found in the database), 38 microRNAs (miRNA), and 111 long non-coding RNAs (lncRNA) (Fig. 6). In the network, 6 miRNAs regulated by 19 lncRNAs could competitively bind with GYS1 mRNA. 16 miRNAs regulated by 28 lncRNAs bind with MYH10 mRNA. 12 miRNAs regulated by 59 lncRNAs were identified to target to CAPZB mRNA. Additionally, 4 miRNAs regulated by 5 lncRNAs were identified to bind with PDLIM1, forming a complex network. (Fig. 6A). Notably, the ceRNA network predicted the regulatory role of RP11-186N15.3 targeted all the five core diagnostic genes simultaneously.

**Fig. 7:**
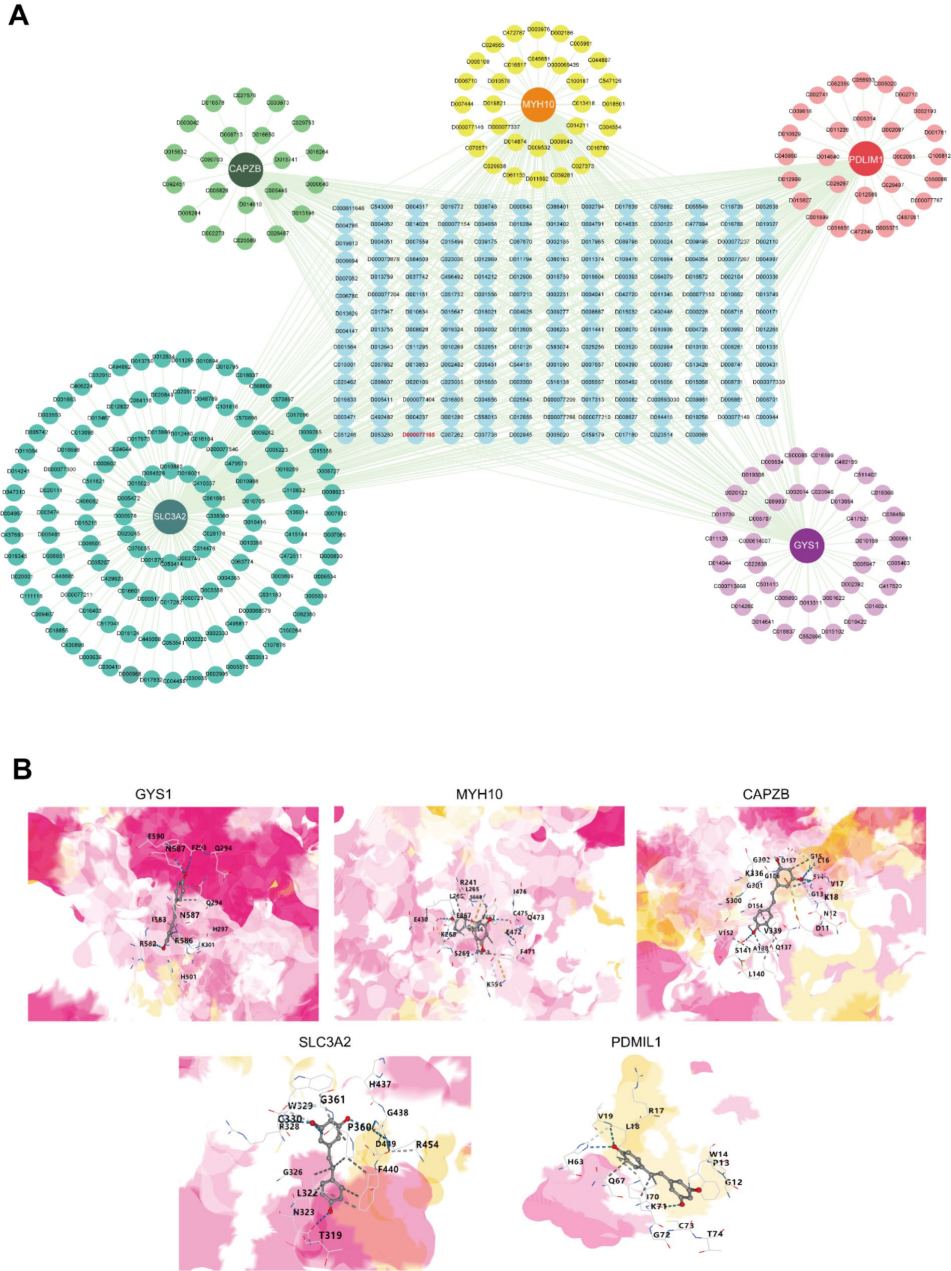
Prediction of chemicals targeting the five core markers. (**A**) Drug-gene network. Chemical ID in red color represents resveratrol. (**B**) Molecular docking schematic diagrams of resveratrol docked with five core markers respectively

Drug prediction targeting the five core diagnostic genes was performed using the CTD database and visualized using the Cytoscape software (Fig. 7A, Supplementary Table S1). Drug testing showed that resveratrol (RSV) could simultaneously act on the five core diagnostic markers with molecular docking conducted with the five markers (Fig. 7B), indicating the potential role of RSV in the treatment of HCM.

### Five core markers were validated in HCM animal model

**Fig. 8:**
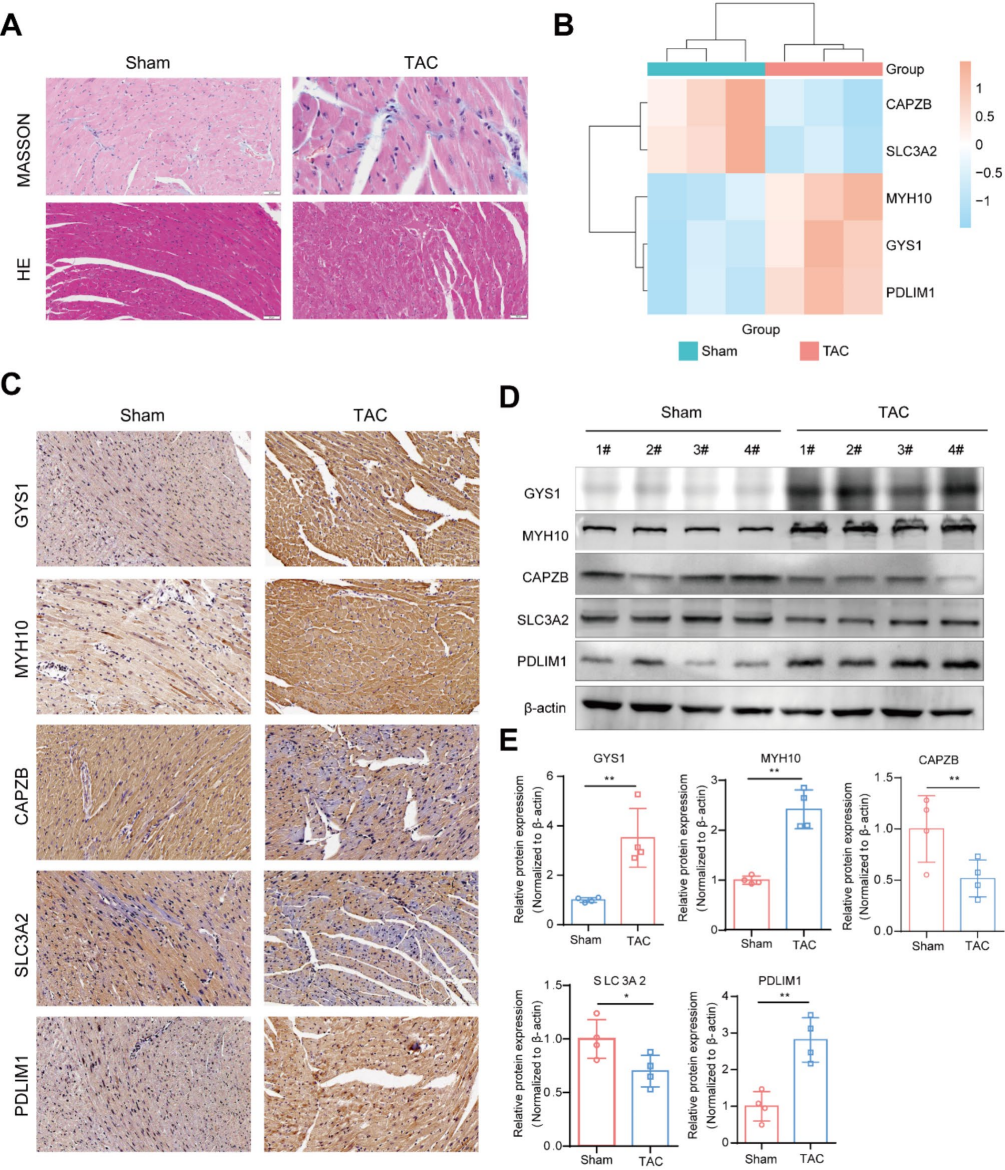
In vivo validation of the five disulfidptosis-related markers in HCM mice. (**A**) Masson and HE staining of control and TAC mice. (**B**) Heatmap of the relative mRNA expressions of *Gys1*, *Myh10*, *Capzb*, *Slc3a2* and *Pdlim1* in control and HCM mice heart tissues (*n* = 3). (**C**) Typical images of immunohistochemical staining of GYS1, MYH10, CAPZB, SLC3A2 and PDLIMI in heart tissues of control and HCM mice. (**D**) Protein expressions of GYS1, MYH10, CAPZB, SLC3A2 and PDLIMI in control and HCM heart tissues detected by Western Blot. (**E**) Quantitative analysis of the Western Blot results. Data are representative of three independent experiments and presented as mean ± SD. Statistical significance was analyzed by unpaired Student’s t-test. ns, no significant, * p< *0.05*, ** p< *0.01*

Finally, we established an HCM mouse model using TAC operation to verify these diagnostic markers in vivo. Animal ultrasound showed that, compared to control, HCM model had increased IVS; d, IVS; s and LVD mass, suggesting left ventricular hypertrophy, and decreased ejection fraction and fractional shortening, indicating left ventricular dysfunction (Supplementary Fig. 2C). In addition, masson and HE staining suggested increased myocardial fibrosis and disordered myocardial alignment in HCM mice (Fig. 8A). These results confirmed the successful establishment of HCM model. Subsequently, we separated the mice’s heart tissue and conducted RNA-seq to validate the differences in the expression of 5 key genes. As shown in Fig. 8B, the mRNA expressions of *Gys1*, *Myh10* and *Pdlim1* were obviously increased while *Slc3A2* and *Capzb* were down-regulated in HCM mice. At the protein level, immunohistochemistry and western blot results showed a significant increase of GYS1, MYH10 and PDLIM1 expressions and a decrease of SLC3A2 and CAPZB exprssions, aligning with previous findings (Fig. 8C-E).

In conclusion, we identified 16 DRGs that are significantly associated with the occurrence of HCM. Among the machine learning models, the SVM model stood out as the optimal model, with five key genes identified as GYS1, MYH10, SLC3A2, CAPZB and PDLIM1. Validation of these core genes using the GSE141910 and GSE160997 datasets showed excellent performance. Then, the ceRNA network was constructed, indicating the regulatory mechanisms of the five core genes. Drug prediction analysis suggested that resveratrol targeting all five markers may have therapeutic benefits for HCM. Importantly, in vivo experiments using the HCM mouse model verified the expression changes of the five markers. Our results provided potential disulfidptosis-related markers for HCM occurrence and suggested a therapeutic effect of resveratrol on HCM.

## Discussion

Hypertrophic cardiomyopathy is a hereditary disease with high clinical incidence. Traditionally, HCM is regarded as the most common single-gene genetic myocardiopathy with the mutation of sarcomere gene (Maron et al. 2022). However, there are still approximately 40% of HCM patients without mutations and the pathogenesis of HCM has not been fully elucidated. Consequently, it is critical to decipher the regulatory mechanisms in the occurrence and development of HCM and identify new biomarkers and therapeutic targets (Maron and Maron 2013). Recent research has highlighted disulfidptosis as a novel form of cell death but its relationship with HCM has not been studied yet. In this study, we demonstrated that disulfidptosis is a critical cell death program in HCM and five related genes (GYS1, MYH10, PDMIL1, SLC3A2, CAPZB) played an important role in the pathological process through comprehensive screening and experiment validation. This research aimed to devise an HCM diagnostic model centered on DRGs and put forward potential therapeutic drugs, offering insights into HCM diagnosis and therapeutic approaches.

Our study identified 16 differentially expressed disulfidptosis-related genes in HCM patients, including upregulation of GYS1, NDUFS1, NDUFA11, DSTN, MYH10, PDLIM1, OXSM and downregulation of SLC3A2, RNP1, ACTIN4, ACTB, CAPZB, FLNA, IQGAP1, MYH9 and TLN1 (Fig. 2). Apart from disulfidptosis, these genes play distinct roles in cardiovascular diseases, so they display different expression patterns in HCM patients. Among these genes, ACTB was reported to be lowly expressed in HCM peripheral blood samples (Feng and Han 2022), which is consistent with our findings. In addition, some literature also evidenced the differential expressions of ACTB in a variety of cardiovascular diseases (Wang et al. 2019; Xue et al. 2018). GYS1, which was increased in HCM patients by our findings, has been reported to be related to heart metabolism and cardiac diseases (Santamans et al. 2021). SLC3A2, which was found to be associated with ferroptosis and atherosclerosis plaque progression (Xiang et al. 2023), was decreased in HCM patients. FLNA, a large actin-binding cytoskeletal protein that is important for cell motility by stabilizing actin networks, has been shown to mediate the progression of myocardial infarction and atherosclerosis (Bandaru et al. 2015, 2019). Interestingly, MYH9 and MYH10 genes encode distinct myosin heavy chain proteins from MYH7 genes, whose mutation would lead to the occurrence of HCM (McKenna and Judge 2021), indicating their potential roles in the occurrence and progression of HCM. Thus, these findings identified the markers as important novel mediators of HCM and potential targets for therapy.

The immune system plays a crucial role in cardiac function, which is significant in HCM. It triggers inflammatory responses and subsequent myocardial repair following injury (Rurik et al. 2021). Further analysis revealed the relationship between immune responses and HCM. 7 of 22 immune cells changed their infiltration pattern in HCM patients, specifically, increased proportion of naïve B cells, regulatory T cells (Tregs), gama delta T cells, resting NK cells and M2 macrophages, and decreased proportion of monocytes and activated dendritic cells. Studies have shown that macrophages play an important role in the initial inflammatory process and subsequent wound healing of myocardial injury (Dick et al. 2019; Jia et al. 2022; Wong et al. 2021). In the early stage of cardiac injury, macrophages are usually polarized to pro-inflammatory M1 type, which accelerates injury by secreting pro-inflammatory cytokines. While in the late stage of injury, M2 macrophages were recruited, which inhibit the expression of collagenase and matrix metalloproteinases by stimulating the synthesis and secretion of extracellular matrix (ECM) proteins, promoting tissue repair (Jia et al. 2022). Both B and T lymphocytes are important in cardiac homeostasis and response to injury. The imbalance of T cell subgroups is closely related to the occurrence and development of myocarditis (Axelrod et al. 2022; Hua et al. 2020; Xia et al. 2020). Especially, Tregs are critical in maintaining immune homeostasis and regulating inflammatory disease progressions (Sakaguchi et al. 2020). It has been widely reported that Tregs have a protective role in heart injury, including myocardial infarction (MI) and myocarditis, by alleviating local inflammation, protecting cardiomyocytes from apoptosis and modulating macrophage differentiation and myofibroblast activation (Xia et al. 2020; Weirather et al. 2014; Sharir et al. 2014; Tang et al. 2012). Thus, the enhanced infiltration of M2 macrophages and Tregs may be a self-protective mechanism in HCM patients.

Moreover, the correlation analysis revealed that disulfidptosis may occur in naïve B cells, activated dendritic cells, neutrophils, activated NK cells, naïve CD4^+^ T cells and CD8^+^ T cells (Fig. 2F). However, research on the role of disulfidptosis in immune cells is limited, mainly focusing on the cancer field. For example, the disulfidptosis-related gene SLC7A11 is proposed to potentially emerge as a new prognostic biomarker for hepatocellular carcinoma (HCC), presenting opportunities for developing personalized cancer immunotherapy strategies (Li et al. 2023). Furthermore, immune checkpoint genes, such as TNFRSF14, TNFRSF4, TNFSF4, BTN2A1, and BTN2A2, are suggested to have a strong correlation with disulfidptosis and may play an essential role in enhancing tumor immunity (Chen et al. 2023a, b). In addition, a group of disulfidptosis-related genes (GYS1, LRPPRC, NDUFA11, OXSM, RPN1, SLC3A2, and SLC7A1) are found to influence the prognosis of bladder urothelial carcinoma via immune cell infiltration (Xin et al. 2023).

Machine learning, an artificial intelligence technology, enables computers to learn laws from data and realize prediction ability. Its principle is to use algorithms and mathematical models to analyze data, identify patterns and trends, and make adjustments to improve accuracy (Greener et al. 2022). Five core DRGs (GYS1, MYH10, PDLIM1, SLC3A2 and CAPZB) were identified by SVM model, which stood out for the best performance and accuracy. Importantly, IHC and WB results of heart tissues from HCM mice were consistent with preliminary bioinformatics analysis, further proving the potential of these genes as diagnostic and therapeutic targets.

Subsequently, drugs targeting these core genes were predicted with CTD database. Mentionably, resveratrol (RSV), a natural polyphenol that can be extracted and purified from wine and grapes, was screened as a chemical that targeted all the five core markers, indicating its potential therapeutic effect on HCM. In fact, the protective effect of RSV on cancer, inflammatory and cardiovascular diseases has been widely reported (Song et al. 2020; Ren et al. 2021). Several studies have shown that cardiomyocyte-specific LKB1 deletion could lead to hypertrophic cardiomyopathy (Molaei et al. 2022; Ikeda et al. 2009). Interestingly, the cardioprotective effect of RSV was reported to be associated with LKB1 activity (Huang et al. 2021), exhibiting the potential therapeutic function of RSV on HCM. Further studies should be focused on the specific effects and underlying mechanisms of RSV in HCM treatments.

However, our study still has some limitations. Firstly, more clinical data on HCM patients are needed to validate the expression levels of DRGs. Secondly, increasing the sample size is essential to improve the accuracy of the DRGs-based model. Last but not least, additional experiments are needed to evaluate the therapeutic effect of resveratrol on HCM.

## Electronic supplementary material

Below is the link to the electronic supplementary material.

Supplementary Material 1

Supplementary Material 2

Supplementary Material 3

Supplementary Material 4

## Data Availability

Data is provided within the manuscript or supplementary information files.

## References

[CR23] Agarwal V et al. Predicting effective microRNA target sites in mammalian mRNAs. Elife, 2015. 4. 10.7554/eLife.05005 PMC4532895 26267216

[CR6] Amgalan D, Chen Y, Kitsis RN. Death receptor signaling in the heart: cell survival, apoptosis, and Necroptosis. Circulation. 2017;136(8):743–6. 28827219 10.1161/CIRCULATIONAHA.117.029566 PMC5657500

[CR17] Asadi S, Roshan S, Kattan MW. Random forest swarm optimization-based for heart diseases diagnosis. J Biomed Inf. 2021;115:103690. 10.1016/j.jbi.2021.103690 33540075

[CR42] Axelrod ML, et al. T cells specific for α-myosin drive immunotherapy-related myocarditis. Nature. 2022;611(7937):818–26. 36385524 10.1038/s41586-022-05432-3 PMC9930174

[CR36] Bandaru S, et al. Deficiency of filamin A in endothelial cells impairs left ventricular remodelling after myocardial infarction. Cardiovasc Res. 2015;105(2):151–9. 25344364 10.1093/cvr/cvu226

[CR35] Bandaru S, et al. Targeting Filamin A reduces macrophage activity and atherosclerosis. Circulation. 2019;140(1):67–79. 31014088 10.1161/CIRCULATIONAHA.119.039697

[CR22] Chen Y, Wang X. miRDB: an online database for prediction of functional microRNA targets. Nucleic Acids Res. 2020;48(D1):D127–31. 31504780 10.1093/nar/gkz757 PMC6943051

[CR5] Chen X, et al. Copper homeostasis and copper-induced cell death in the pathogenesis of cardiovascular disease and therapeutic strategies. Cell Death Dis. 2023a;14(2):105. 36774340 10.1038/s41419-023-05639-w PMC9922317

[CR50] Chen Y, et al. A novel disulfidptosis-related immune checkpoint genes signature: forecasting the prognosis of hepatocellular carcinoma. J Cancer Res Clin Oncol. 2023b;149(14):12843–54. 37462769 10.1007/s00432-023-05076-4 PMC10587022

[CR25] Davis AP, et al. Comparative toxicogenomics database (CTD): update 2021. Nucleic Acids Res. 2021;49(D1):D1138–43. 33068428 10.1093/nar/gkaa891 PMC7779006

[CR7] Del Re DP, et al. Fundamental mechanisms of regulated cell death and implications for Heart Disease. Physiol Rev. 2019;99(4):1765–817. 31364924 10.1152/physrev.00022.2018 PMC6890986

[CR39] Dick SA, et al. Self-renewing resident cardiac macrophages limit adverse remodeling following myocardial infarction. Nat Immunol. 2019;20(1):29–39. 30538339 10.1038/s41590-018-0272-2 PMC6565365

[CR10] Fang X, et al. Loss of Cardiac Ferritin H facilitates Cardiomyopathy via Slc7a11-Mediated ferroptosis. Circ Res. 2020;127(4):486–501. 32349646 10.1161/CIRCRESAHA.120.316509

[CR8] Fang X, et al. The molecular and metabolic landscape of iron and ferroptosis in cardiovascular disease. Nat Rev Cardiol. 2023;20(1):7–23. 35788564 10.1038/s41569-022-00735-4 PMC9252571

[CR30] Feng W, Han S. *lncRNA ADAMTS9-AS1/circFN1 Competitively Binds to miR-206 to Elevate the Expression of ACTB, Thus Inducing Hypertrophic Cardiomyopathy.* Oxid Med Cell Longev, 2022. 2022: p. 1450610. 10.1155/2022/1450610 PMC8989615 35401927

[CR24] Furió-Tarí P, et al. spongeScan: a web for detecting microRNA binding elements in lncRNA sequences. Nucleic Acids Res. 2016;44(W1):W176–80. 27198221 10.1093/nar/gkw443 PMC4987953

[CR20] Gold C, Holub A, Sollich P. Bayesian approach to feature selection and parameter tuning for support vector machine classifiers. Neural Netw. 2005;18(5–6):693–701. 16111861 10.1016/j.neunet.2005.06.044

[CR52] Greener JG, et al. A guide to machine learning for biologists. Nat Rev Mol Cell Biol. 2022;23(1):40–55. 34518686 10.1038/s41580-021-00407-0

[CR26] Guo J, et al. Canopy 2 attenuates the transition from compensatory hypertrophy to dilated heart failure in hypertrophic cardiomyopathy. Eur Heart J. 2015;36(37):2530–40. 26160001 10.1093/eurheartj/ehv294 PMC4589657

[CR21] Harrell JF. *rms: Regression Modeling Strategies*. 2023; R package version 6.8-0:[ https://github.com/harrelfe/rms, https://hbiostat.org/R/rms/

[CR43] Hua X, et al. Single-cell RNA sequencing to dissect the Immunological Network of Autoimmune Myocarditis. Circulation. 2020;142(4):384–400. 32431172 10.1161/CIRCULATIONAHA.119.043545

[CR57] Huang Y, et al. Resveratrol-induced Sirt1 phosphorylation by LKB1 mediates mitochondrial metabolism. J Biol Chem. 2021;297(2):100929. 34216621 10.1016/j.jbc.2021.100929 PMC8326426

[CR56] Ikeda Y, et al. Cardiac-specific deletion of LKB1 leads to hypertrophy and dysfunction. J Biol Chem. 2009;284(51):35839–49. 19828446 10.1074/jbc.M109.057273 PMC2791013

[CR3] Jia D, et al. Interleukin-35 promotes macrophage survival and improves Wound Healing after myocardial infarction in mice. Circ Res. 2019;124(9):1323–36. 30832557 10.1161/CIRCRESAHA.118.314569

[CR40] Jia D, et al. Cardiac Resident macrophage-derived Legumain improves Cardiac Repair by promoting clearance and degradation of apoptotic cardiomyocytes after myocardial infarction. Circulation. 2022;145(20):1542–56. 35430895 10.1161/CIRCULATIONAHA.121.057549

[CR18] Kanda E et al. Machine Learning models Predicting Cardiovascular and renal outcomes and mortality in patients with Hyperkalemia. Nutrients, 2022. 14(21). 10.3390/nu14214614 PMC9658112 36364890

[CR49] Li XM, et al. Identification of disulfidptosis-related genes with immune infiltration in hepatocellular carcinoma. Heliyon. 2023;9(8):e18436. 37520990 10.1016/j.heliyon.2023.e18436 PMC10382636

[CR11] Liu X, et al. Actin cytoskeleton vulnerability to disulfide stress mediates disulfidptosis. Nat Cell Biol. 2023;25(3):404–14. 36747082 10.1038/s41556-023-01091-2 PMC10027392

[CR27] Machesky LM. Deadly actin collapse by disulfidptosis. Nat Cell Biol. 2023;25(3):375–6. 36918690 10.1038/s41556-023-01100-4

[CR19] Mahmoudi Z, et al. Heart failure: a prevalence-based and model-based cost analysis. Front Cardiovasc Med. 2023;10:1239719. 38107256 10.3389/fcvm.2023.1239719 PMC10722181

[CR1] Marian AJ, Braunwald E. Hypertrophic cardiomyopathy: Genetics, Pathogenesis, clinical manifestations, diagnosis, and Therapy. Circ Res. 2017;121(7):749–70. 28912181 10.1161/CIRCRESAHA.117.311059 PMC5654557

[CR29] Maron BJ, Maron MS. Hypertrophic Cardiomyopathy Lancet. 2013;381(9862):242–55. 22874472 10.1016/S0140-6736(12)60397-3

[CR14] Maron BA, et al. Individualized interactomes for network-based precision medicine in hypertrophic cardiomyopathy with implications for other clinical pathophenotypes. Nat Commun. 2021;12(1):873. 33558530 10.1038/s41467-021-21146-y PMC7870822

[CR28] Maron BJ, et al. Diagnosis and evaluation of hypertrophic cardiomyopathy: JACC state-of-the-art review. J Am Coll Cardiol. 2022;79(4):372–89. 35086660 10.1016/j.jacc.2021.12.002

[CR37] McKenna WJ, Judge DP. Epidemiology of the inherited cardiomyopathies. Nat Rev Cardiol. 2021;18(1):22–36. 32895535 10.1038/s41569-020-0428-2

[CR55] Molaei A, et al. LKB1: an emerging therapeutic target for cardiovascular diseases. Life Sci. 2022;306:120844. 35907495 10.1016/j.lfs.2022.120844

[CR12] Rabinovich-Nikitin I, Kirshenbaum LA. YAP/TFEB pathway promotes autophagic cell death and hypertrophic cardiomyopathy in lysosomal storage diseases. J Clin Invest, 2021. 131(5). 10.1172/JCI146821 PMC7919707 33645545

[CR54] Ren B, et al. Resveratrol for cancer therapy: challenges and future perspectives. Cancer Lett. 2021;515:63–72. 34052324 10.1016/j.canlet.2021.05.001

[CR15] Ritchie ME, et al. Limma powers differential expression analyses for RNA-sequencing and microarray studies. Nucleic Acids Res. 2015;43(7):e47. 25605792 10.1093/nar/gkv007 PMC4402510

[CR38] Rurik JG, Aghajanian H, Epstein JA. Immune cells and immunotherapy for Cardiac Injury and Repair. Circ Res. 2021;128(11):1766–79. 34043424 10.1161/CIRCRESAHA.121.318005 PMC8171813

[CR45] Sakaguchi S, et al. Regulatory T cells and human disease. Annu Rev Immunol. 2020;38:541–66. 32017635 10.1146/annurev-immunol-042718-041717

[CR33] Santamans AM, et al. 38γ and p38δ regulate postnatal cardiac metabolism through glycogen synthase 1. PLoS Biol. 2021;19(11):e3001447. 34758018 10.1371/journal.pbio.3001447 PMC8612745

[CR47] Sharir R, et al. Experimental myocardial infarction induces altered regulatory T cell hemostasis, and adoptive transfer attenuates subsequent remodeling. PLoS ONE. 2014;9(12):e113653. 25436994 10.1371/journal.pone.0113653 PMC4249913

[CR53] Song JY et al. Influence of Resveratrol on the Cardiovascular Health effects of chronic kidney disease. Int J Mol Sci, 2020. 21(17). 10.3390/ijms21176294 PMC7504483 32878067

[CR13] Tan WLW, et al. Epigenomes of human hearts reveal New Genetic variants relevant for Cardiac Disease and phenotype. Circ Res. 2020;127(6):761–77. 32529949 10.1161/CIRCRESAHA.120.317254 PMC10935612

[CR48] Tang TT, et al. Regulatory T cells ameliorate cardiac remodeling after myocardial infarction. Basic Res Cardiol. 2012;107(1):232. 22189560 10.1007/s00395-011-0232-6

[CR2] Tuohy CV, et al. Hypertrophic cardiomyopathy: the future of treatment. Eur J Heart Fail. 2020;22(2):228–40. 31919938 10.1002/ejhf.1715

[CR4] Veselka J, et al. Sex-related differences in outcomes of Alcohol Septal ablation for hypertrophic obstructive cardiomyopathy. JACC Cardiovasc Interv. 2021;14(12):1390–2. 34167687 10.1016/j.jcin.2021.03.066

[CR31] Wang W, et al. Integration of Gene expression Profile Data of Human Epicardial adipose tissue from Coronary Artery Disease to Verification of hub genes and pathways. Biomed Res Int. 2019;2019:p8567306. 10.1155/2019/8567306 PMC6900948 31886261

[CR46] Weirather J, et al. Foxp3 + CD4 + T cells improve healing after myocardial infarction by modulating monocyte/macrophage differentiation. Circ Res. 2014;115(1):55–67. 24786398 10.1161/CIRCRESAHA.115.303895

[CR9] Weng L, et al. TGF-β1/SMAD3 regulates programmed cell death 5 that suppresses Cardiac Fibrosis Post-myocardial Infarction by inhibiting HDAC3. Circ Res. 2023;133(3):237–51. 37345556 10.1161/CIRCRESAHA.123.322596

[CR16] Wilkerson MD, Hayes DN. ConsensusClusterPlus: a class discovery tool with confidence assessments and item tracking. Bioinformatics. 2010;26(12):1572–3. 20427518 10.1093/bioinformatics/btq170 PMC2881355

[CR41] Wong NR, et al. Resident cardiac macrophages mediate adaptive myocardial remodeling. Immunity. 2021;54(9):2072. –2088.e7. 34320366 10.1016/j.immuni.2021.07.003 PMC8446343

[CR44] Xia N, et al. A Unique Population of Regulatory T Cells in Heart Potentiates Cardiac Protection from myocardial infarction. Circulation. 2020;142(20):1956–73. 32985264 10.1161/CIRCULATIONAHA.120.046789

[CR34] Xiang P, et al. Metabolite Neu5Ac triggers SLC3A2 degradation promoting vascular endothelial ferroptosis and aggravates atherosclerosis progression in ApoE(-/-)mice. Theranostics. 2023;13(14):4993–5016. 37771765 10.7150/thno.87968 PMC10526676

[CR51] Xin S, et al. A novel model based on disulfidptosis-related genes to predict prognosis and therapy of bladder urothelial carcinoma. J Cancer Res Clin Oncol. 2023;149(15):13925–42. 37541976 10.1007/s00432-023-05235-7 PMC11797905

[CR32] Xue J, et al. Exploring miRNA-mRNA regulatory network in cardiac pathology in na(+)/H(+) exchanger isoform 1 transgenic mice. Physiol Genomics. 2018;50(10):846–61. 30029588 10.1152/physiolgenomics.00048.2018 PMC6230871

