# Supplementary figures and images for: Identification and validation of differentially expressed disulfidptosis-related genes in hypertrophic cardiomyopathy
Source: Mol Med. 2024 Dec 19;30:249. doi: 10.1186/s10020-024-01024-1 (PMC11660498; doi:10.1186/s10020-024-01024-1)

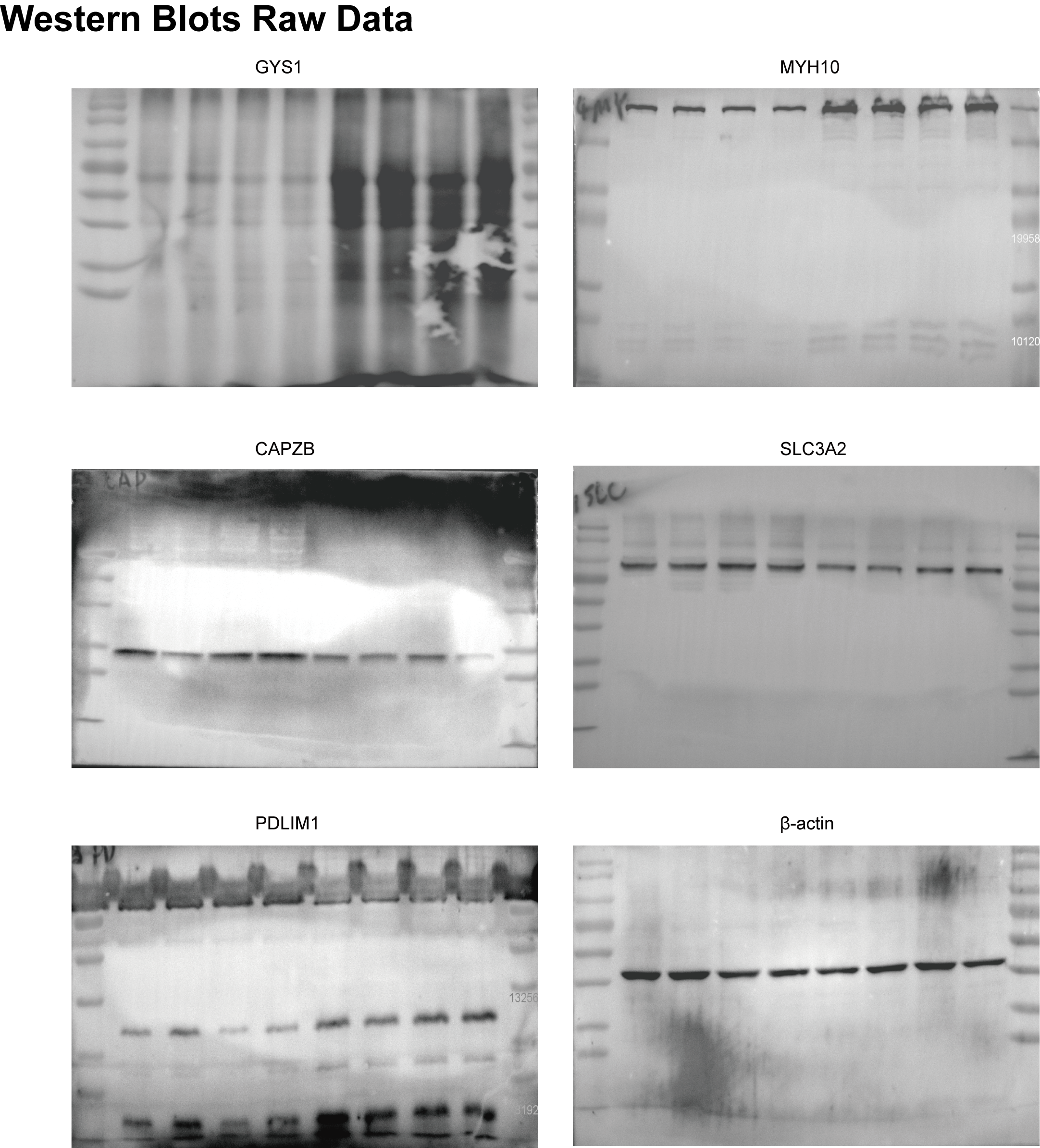

Supplement: Supplementary file 1 — Supplementary Material 1 [file 10020_2024_1024_MOESM1_ESM.tif]

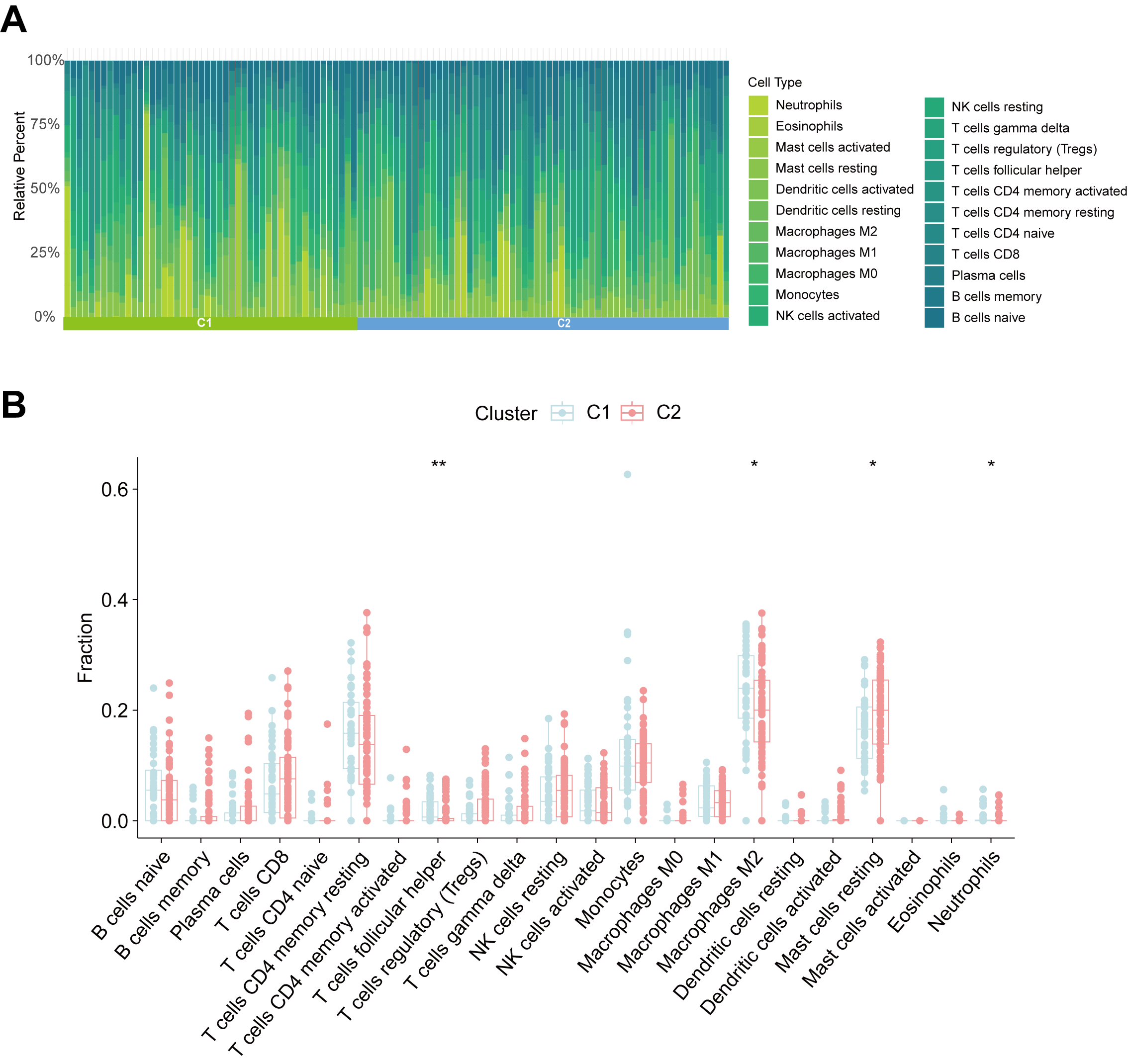

Supplement: Supplementary file 2 — Supplementary Material 2 [file 10020_2024_1024_MOESM2_ESM.tif]

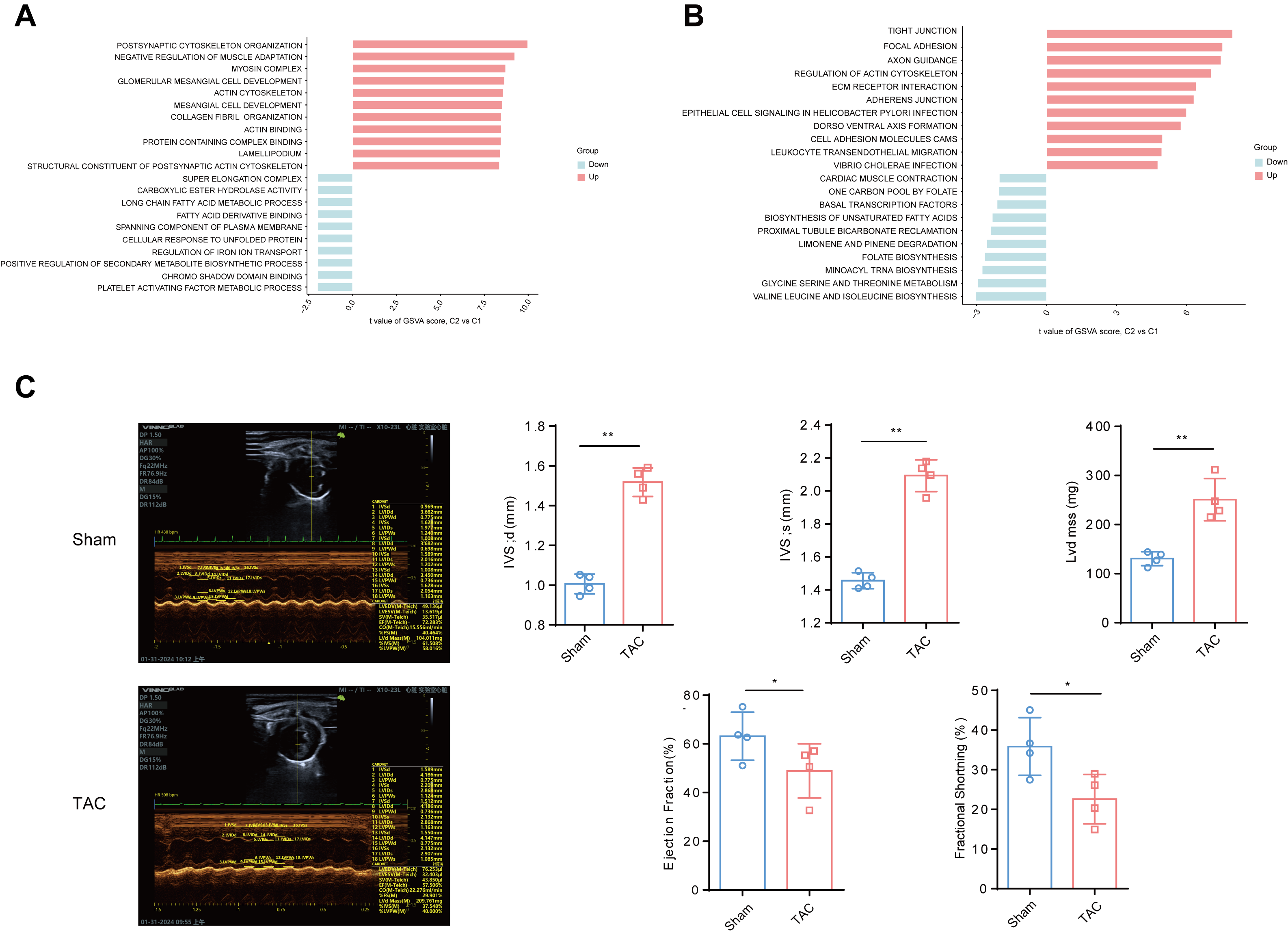

Supplement: Supplementary file 4 — Supplementary Material 4 [file 10020_2024_1024_MOESM4_ESM.tif]
